# Case Report of a Mare Diagnosed with a Metastatic Mammary Carcinoma after the Excision of a Recurrent Intraocular Neuroepithelial Tumor

**DOI:** 10.3390/ani10122409

**Published:** 2020-12-16

**Authors:** Ginevra Brocca, Cinzia Centelleghe, Elisa Padoan, Riccardo Stoppini, Chiara Giudice, Massimo Castagnaro, Valentina Zappulli

**Affiliations:** 1 Department of Comparative Biomedicine and Food Science, University of Padua, Legnaro, 35020 Padua, Italy; cinzia.centelleghe@gmail.com (C.C.); massimo.castagnaro@unipd.it (M.C.); valentina.zappulli@unipd.it (V.Z.); 2 Equine Veterinary Practitioner, Chioggia, 30015 Venice, Italy; elisa.padoan.vet@gmail.com; 3 Consultant in Equine Ophthalmology, Lonato del Garda, 25017 Brescia, Italy; stopric@gmail.com; 4 Department of Veterinary Medicine, University of Milan, Lodi, 26900 Lodi, Italy; chiara.giudice@unimi.it

**Keywords:** eye, histology, immunohistochemistry, mammary carcinoma, mammary gland, metastasis, neuroepithelial tumor, oncology-equine

## Abstract

**Simple Summary:**

This case report describes the unusual presentation of two rare tumors in a mare diagnosed over a short period of time (9 months), affecting the eye and the mammary gland, with a relapse of the ocular tumor presenting histological features that cannot be attributed to any specific neoplastic entity. In the study, we describe the in vivo diagnosis and treatment of the neuroepithelial intraocular tumor and the post mortem detection of a metastatic malignant mammary carcinoma.

**Abstract:**

A 24-year-old Irish Cob mare was presented with a peripheral iris mass, which was surgically resected and diagnosed as an undifferentiated neuroepithelial tumor. A few months later, a relapse occurred with histological features characterized by a more solid appearance and squamous differentiation. Subsequently, the mare was presented with rapidly spreading multiple subcutaneous masses and, at the onset of neurological signs, was humanely euthanized and subjected to a complete post mortem examination. The necropsy confirmed the presence of numerous widespread masses in the subcutaneous tissue, several internal organs, and mammary gland. Histological and immunohistochemical (IHC) examinations were performed on all masses, allowing the diagnosis of mammary carcinoma with several visceral and subcutaneous metastases. Considering the post mortem findings, the second intraocular mass was submitted to histological and IHC re-evaluation to differentiate it from an intraocular metastasis of the mammary carcinoma. The results of the histological and IHC analyses confirmed the diagnosis of neuroepithelial tumor relapse. This is the first case of a metastatic mammary carcinoma concurrent with a recurrent intraocular neuroepithelial tumor in a mare. This case was a challenge for both clinicians and pathologists involved and highlighted the importance of post mortem and IHC evaluations.

## 1. Introduction

Multiple primary neoplasms (i.e., two or more malignant tumors in one patient) are reported in human medicine and are considered uncommon, though their incidence is increasing [1]. Females are at higher risk in such cases, and the most affected organ is the breast. Generally, in human patients with multiple cancers, more attention is drawn to the first diagnosed neoplasia, and the co-existence of a second affection is often overlooked [1]. Similar cases are even less common in veterinary medicine. Some reports exist in dogs, while only a few dated reports are available regarding horses [2].

Cutaneous tumors, including sarcoids, melanoma, squamous cell carcinoma, mast cell tumor, and lymphoma, are among the most commonly diagnosed neoplasms in equids [3]. The most frequently reported neoplasm of internal organs is the granulosa cell tumor of the ovary. Oncology is still developing in equine medicine; therefore, the most common therapeutic approach to tumors is generally limited to the surgical removal or debulking of the mass [3].

This paper details an unusual case of two distinct neoplasms occurring in the same mare at different times; one recurrent intraocular neuroepithelial tumor and one metastasizing mammary carcinoma.

This case presents an example of the challenges that equine practitioners face when dealing with oncological diseases in horses. It also highlights the importance of collaboration with veterinary pathologists since only the post mortem examination allowed the detection of the site of the primary neoplasia and all sites of metastasis. Moreover, without an accurate IHC characterization, the intraocular tumor would have remained undiagnosed.

## 2. Materials, Methods, and Results

### 2.1. Case Description and In Vivo Investigations

At the end of January 2017, a 24-year-old, non-pregnant and non-lactating Irish Cob mare was presented to the attending veterinarian with blepharospasm and epiphora affecting the right eye.

**Figure 1:**
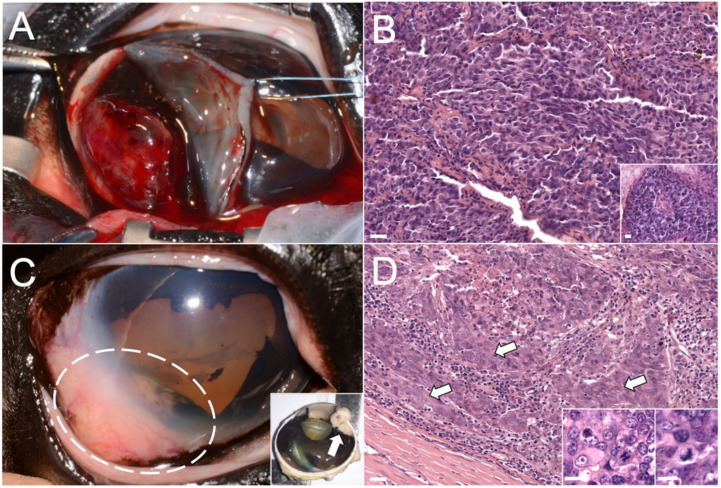
Photographs and photomicrographs of the masses that affected the right eye. (**A**) Gross picture of the primary intraocular mass during the sector iridectomy. (**B**) Photomicrograph of the primary intraocular mass, hematoxylin and eosin (HE) stain; bar = 10 µm. Inset: detail of the pseudo-rosette formation. HE stains, bar = 10 µm. (**C**) Gross picture of the second intraocular mass in the same intraocular location (dashed circle), sagittal section (after enucleation) in the inset (arrow). (**D**) Photomicrograph of the second intraocular neoplasia, squamous differentiation indicated by arrows. HE stain; bar = 10 µm. Inset: detail of bizarre mitotic figures. HE stains, bar = 5 µm.

Ocular ultrasound showed a well-defined, 1.2 cm large intraocular mass, which led to a preliminary diagnosis of iris neoplasia with secondary uveitis. A complete blood cell count and serum biochemistry analysis were performed, and all values fell within reference ranges. The mass was removed (Figure 1A) in February, under general anesthesia, through a sector iridectomy using a laser diode. Damage to the lens and corneal endothelium was prevented using intraocular viscoelastic material. The mass was sent to the veterinary histopathology diagnostic service of the Department of Veterinary Medicine of the University of Milan.

The mass was fixed in 10% buffered formalin for over 72 h. The paraffin-embedded block was cut at 4 µm-thick sections and routinely stained with hematoxylin and eosin (HE).

Histologically, the mass was not encapsulated and poorly demarcated, growing on the posterior surface of the iris and multifocally infiltrating the surrounding stroma.

The neoplastic population was composed of polygonal cells arranged in cords and small islands, multifocally organized in small tubules and pseudo-rosettes (Figure 1B), and associated with moderate fibrovascular stroma, with occasional lacunae containing amorphous eosinophilic material or red blood cells. Neoplastic cells were characterized by a moderate amount of finely granular and eosinophilic cytoplasm, a high nucleus/cytoplasm ratio, vesicular and round nuclei with the often visible and prominent single nucleolus. Occasionally, a mild amount of cytoplasmic melanic pigment was also present. Anisocytosis and anisokaryosis were moderate, and the mitotic count ranged from 0 to 3 mitoses per high-power field (hpf; 0.237 mm^2^). To rule out other potential differential diagnoses, including uveal melanoma, medulloepithelioma, and metastatic lymphosarcoma [4], immunohistochemical (IHC) analysis was performed.

Immunohistochemical staining was performed using an automatic immunostainer (Ventana Benchmark GX, Roche Diagnostic GmbH, Mannheim, Germany) and the special DAB kit (ultraViews Universal DAB Kit, Roche Diagnostic GmbH, Mannheim, Germany). The expression of the following antigens was evaluated with anti-human antibodies (Abs): panCK, vimentin, neuron-specific enolase (NSE), and melanoma-associated antigen (PNL2).

Neoplastic cells showed to be sparsely positive (15–20%) for panCK, vimentin, and NSE, and negative for PNL2. The mass was diagnosed as a poorly differentiated tumor with an immunophenotype consistent with a primary neuroepithelial tumor.

In May, the mare showed renewed discomfort to the right eye without any other clinical abnormalities. A new ophthalmic examination showed a recurrent intraocular mass in the same location, involving the anterior uvea and limbal sclera (Figure 1C). At this time, the eye was enucleated, and an intraorbital silicone prosthesis was applied. Grossly, a neoplastic mass (1.3 × 1.0 × 0.8 cm) involving the iris root, the iridocorneal angle, and extending to the perilimbal sclera, causing alteration of eye profile, was detected (Figure 1C—insets).

At histological examination, the infiltrative mass was composed of polygonal cells arranged in closely packed solid sheets, showing multifocally squamous differentiation, high mitotic rate and bizarre mitotic figures (Figure 1D—inset). Although the site of origin was consistent with a recurrence of the previous intraocular tumor, histopathology indicated that the new mass was an intraocular carcinoma characterized by more malignant morphological features as compared to what was considered the primary mass (Figure 1D).

The mare recovered well from both surgeries and was again used as a leisure horse. In October, the mare was presented with a grade 3 (3 out of 5, American Association of Equine Practitioners, AAEP, grading) lameness affecting the right pelvic limb. A complete lameness examination was performed, including flexion tests of the right pelvic limb joints and intra-articular anesthesia. The lameness was more severe on the left-hand trot, and the stifle flexion test caused a worsening of it. An intra-articular injection (triamcinolone 14 mg and hyaluronic acid 40 mg/mL) of the right medial femoral-patellar joint was performed, and no abnormalities were found during the sterile scrub procedure. The lameness improved (1 out of 5 AAEP grading) after treatment. Mild weight loss (body condition score 2.5/5) was investigated by complete blood work and serum biochemistry, but all values were within reference ranges. In November, scattered subcutaneous masses were noticed, mainly developing on the back and flanks and rapidly appearing also in the hips. At the same time, the mare started showing neurological deficits, such as ataxia of all four limbs and mild shivers. Although a neoplastic disease was suspected, it was not possible to diagnose because, due to the rapid worsening of the clinical signs, the mare was humanely euthanized at the end of November. Following euthanasia, the mare was sent to the Department of Comparative Biomedicine and Food Science (BCA) of the University of Padua for a full post mortem examination.

The horse owners consented to the use of the derived materials and tissues for research purposes upon submission of the carcass to the facilities of the Veterinary Histopathology Diagnostic Service of the University of Milan and the Veterinary Necropsy Service of the University of Padua.

**Figure 2:**
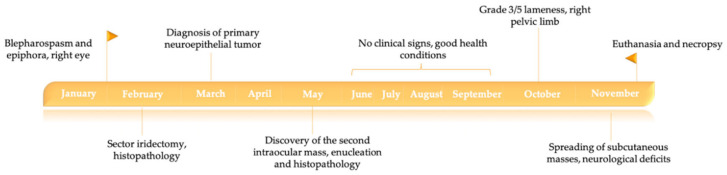
Timeline of the case history.

The chronological order of the events is represented in Figure 2.

### 2.2. Post Mortem Examinations

**Figure 3:**
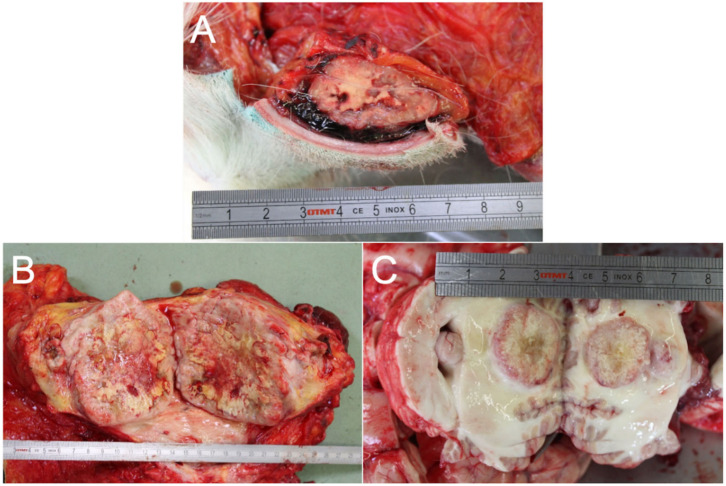
Photographic report produced during the necropsy. (**A**) Gross picture of one of the multiple subcutaneous masses. (**B**) Gross pictures of the mass affecting the right mammary gland, 15 cm in diameter. (**C**) Cerebellar localization of one of the multiple masses detected.

Upon examination, the gross findings confirmed the presence of multifocal masses involving the subcutis (Figure 3A), mainly localized on the back, the flanks, and the hind limbs. A mass approximately 15 cm in diameter was also observed on the right mammary gland (Figure 3B). Additionally, nodular lesions, ranging from 1 to 6 cm in diameter, were detected in numerous internal organs, including the cerebellum (Figure 3C), heart, lungs, liver, pancreas, parotid glands, intestinal wall, adrenal glands, muscles, and lymph nodes. All the nodules were grossly yellow-white, well-demarcated, round to oval, a firm with occasional central areas of necrosis (more represented in the mammary mass).

Samples from all the grossly affected tissues (mammary gland, subcutis, cerebellum, heart, lungs, liver, pancreas, parotid glands, adrenal glands, lymph nodes, and intestine) were processed for histology and immunohistochemistry as previously described and stained with HE for routine microscopic examination.

**Figure 4:**
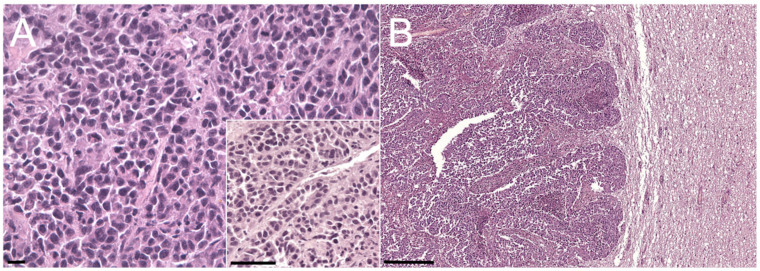
Photomicrographs of sections of the ocular, mammary, and cerebellar masses. (**A**) Primary mammary gland tumor. HE stain; bar = 10 µm. Inset: detail of the tubular structure. HE stain; bar = 25 µm. (**B**) Cerebellar metastasis. HE stain; bar = 100 µm.

Histologically, all the neoplastic masses (Figure 4A,B illustrates the mammary and cerebellar masses, respectively) showed discrete, non-encapsulated, well-demarcated, infiltrative, solid lesions characterized by a dense cellular population. Neoplastic cells were organized in solid sheets with occasional formation of tubules (Figure 4A—inset), admixed with abundant fibrovascular stroma and desmoplasia (scirrhous proliferation). The neoplastic population was characterized by 15–25 µm polygonal well-discernible cells with abundant, homogeneous, moderately eosinophilic cytoplasm. Nuclei were round, 8–12 µm in diameter, hyperchromatic, central to paracentral, with 1 to 3 evident nucleoli and finely stippled chromatin. Anisocytosis and anisokaryosis were moderate, and the mitotic count ranged from 5 to 6 mitoses per hpf (0.237 mm^2^). Occasionally, subcutaneous masses showed more anaplastic areas with highly pleomorphic cells arranged in solid to discrete patterns. Multifocal areas of extensive necrosis were evident, and invasion of blood vessels was frequently observed, mainly at the periphery of the masses. Based on the size and histopathological features, the mammary mass was considered the primary neoplasia associated with widespread metastases.

Serial sections of the mammary gland, lungs, cerebellum, and subcutaneous masses underwent IHC analysis as previously described. The expression of the panCK, CK14, CK5/6, p63, vimentin, calponin, estrogen receptor (ER), progesterone receptor (PR), and NSE antigens was evaluated with anti-human Abs, and the relevant details are provided in Table 1. Tissue sections obtained from the second intraocular mass, retrieved from the archive, were also tested with the same Abs-panel by means of IHC. Due to the small size of the original biopsy, tissue from the first intraocular mass was no longer available for further analysis.

**Figure 5:**
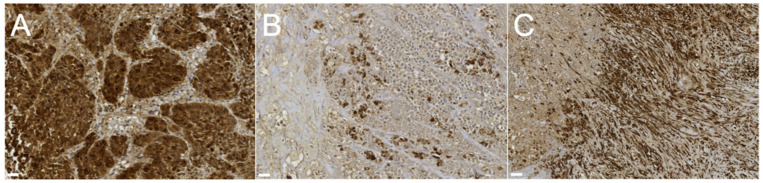
Photomicrographs of IHC-stained sections of the primary mammary gland tumor. (**A**) Strong diffuse positivity of the neoplastic parenchyma for panCK antibody (Ab); bar = 10 µm. (**B**) Strong multifocal positivity of the neoplastic parenchyma for CK 5–6 Ab, bar = 10 µm. (**C**) Strong diffuse positivity of the fibrovascular stroma for vimentin Ab; bar = 20 µm.

In both the primary mammary tumor and metastases (lung, cerebellum, and subcutis), strong cytoplasmic staining was evident for panCK (Figure 5A), pointing towards an epithelial nature for this neoplasia [5,6]. Additionally, the mammary tumor showed strong positivity for both CK14 and CK5-6 (Figure 5B) in a subset of cells; both markers are specific for basal and/or myoepithelial differentiation in mammary tumors [5,6], which is associated with more aggressive behavior of the tumor and a worse prognosis in dogs [6]. Positivity for calponin and vimentin was detected only in the stroma (Figure 5C), implying the tumor was a simplex carcinoma. NSE and p63 were negative in all the lesions. p63, in particular, is a transcription factor: in mammary tumors, a reduction or the loss of its expression is detectable in invasive tumors.

PR expression was negative in the primary mammary tumor and mildly positive in 16% of the cells in the metastases. All the antibodies tested positively cross-reacted with the positive control and with equine tissues except for the anti-ER antibody, which did not cross-react with equine tissues.

The second intraocular mass showed strong positivity in approximately 100% of cells for NSE, panCK, and vimentin, closely resembling the phenotype of the first intraocular mass. It was also strongly positive for CK14 (in approximately 30% of cells), and CK5/6 (in approximately 5% of cells), and negative for calponin, PR, and p63.

The IHC results are summarized in Table 1.

## 3. Discussion

Mammary neoplasms in mares are considered a rare event, usually documented via case reports [7,8,9] or small case series [10], with less than 30 tumors reported so far (prevalence rate of 0.11%) [11]. They are usually malignant, with the unique case of a benign adenoma reported by Spadari and colleagues in 2008 [12]. Mammary neoplasia in mares is commonly misdiagnosed as mastitis [9,10,13,14,15], but in our case, no specific clinical signs of mammary gland involvement were detected. Clinical manifestation, such as weight loss and widespread subcutaneous masses, was compatible with an aggressive tumor, as well as the neurological signs, which could likely be attributed to the acute onset of cerebellar metastasis. Instead, the etiology of unilateral lameness remained unknown.

Histologically, equine primary malignant mammary tumors have been diagnosed as ductal carcinomas [9], solid carcinomas [7,16,17], tubulo-papillary carcinomas [18], papillary ductal adenocarcinomas [14,19,20], or a combination of tubulo-papillary and solid carcinomas [10]. Only one case of micropapillary in situ carcinoma was reported [8]. In our case, the microscopic features of the tumor were the same as those reported in the literature for solid carcinomas.

The majority of equine mammary carcinomas result in high metastatic spread to lymph nodes and visceral organs [8,9,14,15,16,18,19,20], but no previous reports of cerebellar, cardiac, and subcutaneous metastases are present in the literature. In our case, the metastatic behavior of the neoplasia appeared particularly aggressive, and the metastatic lesions appeared more anaplastic, in line with what was previously reported by Kato and colleagues in 1998 [21].

Previous IHC investigations on equine mammary carcinomas generally showed a mild to moderate immunostaining for panCK [10], as reported in our case, highlighting the simple epithelial nature of these lesions in horses. Furthermore, a high proportion (9/13) of mammary tumors in horses are negative or only weakly positive (less than 5% of the neoplastic cells) for the expression of steroid receptors [8,10,12,14,18]. Only 4 out of 13 samples of equine mammary tumors reported so far exhibited convincing ER positivity [8,10,12,14,18], and only one case, to our knowledge, was found positive for both ER and PR [8]. The presence of a few cases reported, coupled with the data presented in this paper, makes the role of sexual hormones unclear in the pathogenesis of equine mammary tumors. However, the presumptive low dependency on sexual hormones could be linked to the highly malignant behavior frequently described [12]. In our case, the anti-ER clone used did not cross-react with equine tissues, and therefore the expression of the ER could not be investigated.

Primary intraocular tumors are a rare event in horses, with medulloepitheliomas (i.e., tumors of primitive neuroepithelium) and uveal melanomas being the most commonly reported neoplasms [4], and a single case of an orbital paraganglioma documented [22]. In the present case, the neoplasm was poorly differentiated, but with the aid of IHC, a poorly pigmented or amelanotic melanoma could be ruled out. Neoplastic cells were positive, although not evenly, for panCK, NSE, and vimentin and this immunophenotype was considered suggestive of a neuroepithelial origin. The second intraocular mass was histologically significantly different, and it was consistent with an infiltrative malignant epithelial tumor with occasional squamous differentiation. During post mortem examination, although considered less likely, an ocular metastasis of the mammary carcinoma could not be excluded. Immunohistochemistry again was pivotal, revealing a recurrence of the neuroepithelial tumor. It was therefore concluded that the mare was affected by two different neoplasms: a recurrent intraocular neuroepithelial tumor and a metastatic mammary carcinoma. Undoubtedly, the latter represented a life-threatening disease, which led to the mare’s death due to multi-visceral involvement. The mare did not present the common clinical signs associated with the few mammary carcinomas reported in the literature, such as local pain and redness, skin ulceration or abundant serosanguinous discharge from teats [15]. Similarly, complete blood count and serum biochemistry did not reflect any signs of systemic disease. In this case, the main diagnostic challenge was posed by the occurrence of two rare neoplastic conditions in a relatively short timeframe, with the metastatic mammary carcinoma being responsible for the poor body condition and the neurological signs described.

Multiple primary tumors in animals have been linked to marked genetic predisposition, and in dogs, a familial syndrome has been hypothesized, particularly in Boxers [2]. In addition, other etiologic factors such as exposure to endogenous or exogenous carcinogens and immunodeficiency have been proposed as contributing causes [23]. However, the lack of specific studies on the etiological factors in multiple equine tumors precludes any speculations nor conclusion on the possible cause in regard to this case.

## 4. Conclusions

Although some clues to the primary cause of health deterioration were evident on clinical examination, such as subcutaneous masses and neurological signs, the primary mammary carcinoma remained undiagnosed, as did visceral organ metastases.

It is noteworthy that also the other primary tumor—an intraocular neuroepithelial neoplasm–showed aggressive behavior with relapse after a few months from surgery.

The uncommon occurrence of multiple primary tumors in a horse can be challenging to diagnose and highlights the importance of post mortem procedures in tumor diagnosis, corroborating the use of IHC techniques in equine pathology as a valid and reliable tool in complex cases.

## Figures and Tables

**Table 1:** List of antibodies applied in the present study.

Antibody	Features	Clone	Dilution	Localization	Tumor Positivity	Metastases Positivity	First Intraocular Mass	Second Intraocular Mass
PanCK	Dako^®^ (Santa Clara, CA, USA) monoclonal	AE1/AE3	1:100	Cytoplasm	75%	60%	15–20%	100%
	mouse anti-human				(strong)	(strong)	(strong)	(strong)
CK 14	Novocastra^®^ (Newcastle, UK) monoclonal	LL002	1:20	Cytoplasm	negative	10%	/	30%
	mouse anti-human					(strong)		(strong)
CK 5/6	Dako^®^ monoclonal	D5/16 B4	1:50	Cytoplasm	15%	19%	/	5%
	mouse anti-human				(strong)	(strong)		(strong)
p63	GeneTex^®^ (Irvine, CA, USA) polyclonal	N2C1	1:200	Nucleus	negative	negative	/	negative
	mouse anti-human							
Vimentin	Dako^®^ monoclonal	V9	1:150	Cytoplasm	stromal		15–20%	100%
	mouse anti-vimentin						(strong)	(strong)
Calponin	Dako^®^ monoclonal	CALP	1:200	Cytoplasm	stromal		/	negative
	mouse anti-human							
ER	Novocastra^®^ monoclonal	6F11	1:40	Cytoplasm	not cross-reactive			
	mouse anti-human							
PR	Ventana^®^ (Tucson, AZ, USA) monoclonal rabbit anti-human	1E2	1:80	Nucleus	negative	16%	/	negative
						(mild)		
NSE	Dako^®^ monoclonal	BBS/NC/VI-H14	1:250	Cytoplasm	negative		15–20%	100%
	mouse anti-human						(strong)	(strong)
PNL2	Santa Cruz Biotech^®^ (Dallas, TX, USA)	sc-59306	1:100	Cytoplasm	/		negative	/
	monoclonal							
	mouse anti-human							
